# Supplementary material for: Discovery and Genomic Function of a Novel Rice Dwarf-Associated Bunya-like Virus
Source: Viruses. 2022 May 29;14(6):1183. doi: 10.3390/v14061183 (PMC9228739; doi:10.3390/v14061183)
Supplement: Supplementary file 1 [file viruses-14-01183-s001.zip › viruses-1729037-supplementary/Supplementary files/supplementary materials-Tables S1,S4,S5,S6 and Figures S1,S2.pdf]

Table S1. Primers used in this study.

Name	Sequence
RDaBV-Detection-F	CTGATCTGGTGAGGTCCATGC
RDaBV-Detection-R	TCAGCCAGTGGACACATAATGTAC
RDaBV-L-1F	GCAATAGTCTCTGATGTTAACTCCTGT
RDaBV-L-2F	CCATGATTTGTTTATCAGCAGCC
RDaBV-L-3F	GAGACAGGTAGTAATCACCAACCTTC
RDaBV-L-1R	CTCGGCATGGTTCAAAGGTTC
RDaBV-L-2R	GAGGGTTTCGAGAGATAGTGATACAG
RDaBV-M-F	GTCAGCAAAAAACTCATGATTGA
RDaBV-M-R	CCTTCCTTACCTGAACAGAC
RDaBV-S-F	GTCAGCAAAAAACTCATGATTGA
RDaBV-S-R	CCTTCCTTACCTGAACAGAC
RDaBV-M-RACE-R1	CTGCATGAATTCAGAATTGTTGACCTG
RDaBV-M-RACE-R2	GGAGAACATCAATGCCCCGAG
RDaBV-M-RACE-R3	CTGAGATAACACTTCCCATTGGAAGAC
RDaBV-M-RACE-F1	GGTGTATTGCAGAGGAAGACTCG
RDaBV-M-RACE-F2	GAGGATGACTCTGGTGATGATGTG
RDaBV-M-RACE-F3	CAGAGGATAGGAGCTATCAATGGTCT
RDaBV-S-RACE-R1	GCTTGAGATCCCCAGGACAC
RDaBV-S-RACE-R2	CCTCCTTTGCCTTGGTTTCTTGT
RDaBV-S-RACE-R3	CTCGTTAACGATTTCCTTTTGGAAGAAC
RDaBV-S-RACE-F1	GCCTCAGGACTTAGTTACTGCATT
RDaBV-S-RACE-F2	GAGATGCACTGCAAGTATTCCAC
RDaBV-S-RACE-F3	GAAAATACCAACGAACGGGTGC
RDaBV-L-RACE-R1	CATAGAAGTGAGAGAAGAAGTCAATGAAG
RDaBV-L-RACE-R2	TGACGGTAGATACTATGAACCACAGG
RDaBV-L-RACE-R3	GATTGCAGAAATCTTCAACTCAACAATGC
RDaBV-L-RACE-F1	GATTTCTCAACCTCTTTGAACTTGAGTGT
RDaBV-L-RACE-F2	CTCATACCCTTGGAACAAACCGTTG
RDaBV-L-RACE-F3	GGGCATACTGATGCTTGGTAGTAGG
RDaBV-NSs-Flag-F	GAGCTCGGTACCCGGGGAATGAAGCTGATCAATTCATATTCAGGCT
RDaBV-NSs-Flag-R	GTCGTCGACTCTAGAGGATAGCAGAACAAGTTCTTCATCAAGCTC
RDaBV-N-Flag-F	GAGCTCGGTACCCGGGGAATGTCTTCAAGCGGAACAG
RDaBV-N-Flag-R	GTCGTCGACTCTAGAGGAATTTGTCTTTGCAACCTGTTG
RDaBV-NS-Flag-F	GAGCTCGGTACCCGGGGAATGTCTCAAGTCAAAATGATTTCAG
RDaBV-NS-Flag-R	GTCGTCGACTCTAGAGGACCTTAGAATGCTGAAAACTGAAG
RDaBV-NSs-GFP-F	GAGCTCGGTACCCGGGGAATGAAGCTGATCAATTCATATTCAGG
RDaBV-NSs-GFP-R	CATGTCGACTCTAGAGGATAGCAGAACAAGTTCTTCATCAAG
RDaBV-N-GFP-F	GAGCTCGGTACCCGGGGAATGTCTTCAAGCGGAACAG
RDaBV-N-GFP-R	CATGTCGACTCTAGAGGAATTTGTCTTTGCAACCTGTTG
RDaBV-NS-GFP-F	GAGCTCGGTACCCGGGGAATGTCTCAAGTCAAAATGATTTCAG
RDaBV-NS-GFP-R	CATGTCGACTCTAGAGGACCTTAGAATGCTGAAAACTGAAG
RDaBV-NSs-pgr106-F	CAGCTAGCATCGATTGGCGCGCCATGAAGCTGATCAATTCATATTCAGG
RDaBV-NSs-pgr106-R	AACCGTTCATCGGCGGTCGACTCATAGCAGAACAAGTTCTTCATCAAG
RDaBV-N-pGR106-F	CAGCTAGCATCGATTGGCGCGCCATGTCTTCAAGCGGAACAG
RDaBV-N-pGR106-R	AACCGTTCATCGGCGGTCGACTCAATTTGTCTTTGCAACCTGTTG
RDaBV-NS-pGR106-F	CAGCTAGCATCGATTGGCGCGCCATGTCTCAAGTCAAAATGATTTCAG
RDaBV-NS-pGR106-R	AACCGTTCATCGGCGGTCGACTCACCTTAGAATGCTGAAAACTGAAG
RBSDV-detection-F	CCGACCAACAATCACTCTGT
RBSDV-detection-R	GGTCAGTTCGTATTCATCGG
SRBSDV-detection-F	TATTCAAAGTTATTTCCGT
SRBSDV-detection-R	ACATGAATAGTTTCAAGT
RDV-detection-F	TACTTTCTCCGGGCTCACAACAGG
RDV-detection-R	CCCCGCAACAGACCGAAACAA

RSV-detection-F	GTCAGACCACGCTCCTTCTTC
RSV-detection-R	GGATATGACTATGTGCATCAC
RRSV-detection-F	ACCGTCGTTGAGCTACCATCCATT
RRSV-detection-R	GGCGGGCCACTCAAACCAT
RGDV-detection-F	CGGAGTATGGGACCAAATGTTCC
RGDV-detection-R	CCGCCTGGTAGCTGGCATATAT
RSMV-detection-F	CTCCAACTATCATCCGCTATGC
RSMV-detection-R	CCATCCGAGATAAGGTCACTGT

Table S4. The BLASTx search using the L segment of RDaBV query.

Description	Scientific Name	family	genus	Query Cover	E value	Per. Ident	Acc. Len	GenBank accession no.	Max Score	Total Score
RNA-dependent RNA polymerase	Botrytis cinerea negative-stranded RNA virus 2	unclassified	unclassified	96%	0	57.86%	2135	QJT73695.1	2527	2527
RNA-dependent RNA polymerase	Erysiphe necator associated negative-stranded RNA virus 8	unclassified	unclassified	96%	0	57.76%	2135	QJW70349.1	2524	2524
RNA-dependent RNA polymerase	Plasmopara viticola lesion associated mycobunyavirales-like virus 8	Discoviridae	Orthodisco virus	96%	0	57.76%	2135	QJX19788.1	2524	2524
RNA-dependent RNA polymerase	Erysiphe necator associated negative-stranded RNA virus 9	unclassified	unclassified	96%	0	54.83%	2139	QJW70358.1	2415	2415
RNA-dependent RNA polymerase	Erysiphe necator associated negative-stranded RNA virus 10	unclassified	unclassified	96%	0	53.73%	2160	QJW70360.1	2312	2312
RNA-dependent RNA polymerase	Erysiphe necator associated negative-stranded RNA virus 18	unclassified	unclassified	96%	0	52.83%	2137	QJW70341.1	2292	2292
RNA-dependent RNA polymerase	Plasmopara viticola lesion associated mycobunyavirales-like virus 9	Discoviridae	Orthodisco virus	97%	0	50.28%	2142	QJX19792.1	2178	2178
RNA-dependent RNA polymerase	Coniothyrium diplodiella negative-stranded RNA virus 1	Discoviridae	Orthodisco virus	97%	0	50.16%	2142	QDB75015.1	2174	2174
RNA-dependent RNA polymerase	Plasmopara viticola lesion associated mycobunyavirales-like virus 10	unclassified	unclassified	83%	0	55.36%	1832	QJX19789.1	2093	2093
RNA-dependent RNA polymerase	Rice Peribunya-like virus 1	Peribunyaviri dae	unclassified	96%	0	47.03%	2137	QKN84393.1	1917	1917
RNA-dependent RNA polymerase	Grapevine-associated negative single-stranded RNA virus 5	unclassified	unclassified	96%	0	46.55%	2138	QXN75415.1	1899	1899
RNA-dependent RNA polymerase	Penicillium roseopurpureum negative ssRNA virus 1	Discoviridae	Orthodisco virus	96%	0	46.36%	2136	AYP71800.1	1862	1862
RNA-dependent RNA polymerase	Fusarium poae negative-stranded-like virus 2	unclassified	unclassified	95%	0	46.55%	2146	QQO58802.1	1857	1857
RNA-dependent RNA polymerase	Fusarium poae negative-stranded virus 2	unclassified	unclassified	96%	0	44.85%	2155	YP_009272912.1	1842	1842
polymerase	Ixodes scapularis associated virus-5	unclassified	unclassified	97%	0	42.33%	2138	AUW34407.1	1590	1590
RNA dependent RNA polymerase	Plasmopara viticola lesion associated mycobunyavirales-like virus 5	unclassified	unclassified	79%	0	47.34%	1791	QGY72642.1	1585	1585
RNA dependent RNA polymerase	Penicillium discovirus	Discoviridae	Orthodisco virus	96%	0	41.48%	2123	AWN00471.1	1584	1584
RNA dependent RNA polymerase	Aspergillus fumigatus negative-stranded RNA virus 1	Discoviridae	Orthodisco virus	96%	0	41.49%	2123	BCH36617.1	1556	1556
RNA dependent RNA polymerase	Erysiphe necator associated negative-stranded RNA virus 4	unclassified	unclassified	93%	0	41.82%	2116	QJW70357.1	1528	1528
RNA dependent RNA polymerase	Erysiphe necator associated negative-stranded RNA virus 3	unclassified	unclassified	95%	0	38.58%	2080	QJW70350.1	1384	1384
RNA dependent RNA polymerase	Plasmopara viticola lesion associated mycobunyavirales-like virus 6	unclassified	unclassified	68%	0	45.69%	1490	QGY72643.1	1285	1285
RNA dependent RNA polymerase	Plasmopara viticola lesion associated mycobunyavirales-like virus 4	Discoviridae	Orthodisco virus	50%	0	53.12%	1113	QGY72641.1	1181	1181

Table S5. The BLASTx search using the M segment of RDaBV query.

Description	Scientific Name	family	genus	Query Cover	E value	Per. Ident	Acc. Len	Accession	Max Score	Total Score
NS	Plasmopara viticola lesion associated mycobunyavirales-like virus 8	Discoviridae	Orthodiscovi rus	89%	4.00E-99	37.77%	489	QNQ74054.1	320	320
NS	Plasmopara viticola lesion associated mycobunyavirales-like virus 4	Discoviridae	Orthodiscovi rus	90%	6.00E-80	33.77%	516	QNQ74053.1	271	271
NS	Plasmopara viticola lesion associated mycobunyavirales-like virus 9	Discoviridae	Orthodiscovi rus	86%	4.00E-59	31.14%	574	QNQ74048.1	217	217
NS1	Coniothyrium diplodiella negative-stranded RNA virus 1	Discoviridae	Orthodiscovi rus	85%	3.00E-51	28.99%	597	QFQ60953.1	196	196
nonstructural protein 1	Penicillium roseopurpureum negative ssRNA virus 1	Discoviridae	Orthodiscovi rus	41%	2.00E-32	33.62%	509	QEQ12678.1	142	142
NS1	Penicillium discovirus	Discoviridae	Orthodiscovi rus	29%	4.00E-14	30.23%	370	AWN00472.1	86.7	86.7

Table S6. The BLASTp search using the N protein of RdaBV query.

Description	Scientific Name	family	genus	Query Cover	E value	Per. Ident	Acc. Len	Accession	Max Score	Total Score
NC	Plasmopara viticola lesion associated mycobunyavirales-like virus 4	Discoviridae	Orthodiscovirus	74%	2.00E-99	53.41%	286	QNQ74050.1	306	306
NC	Plasmopara viticola lesion associated mycobunyavirales-like virus 9	Discoviridae	Orthodiscovirus	68%	3.00E-91	55.38%	261	QNQ74049.1	285	285
NC	Plasmopara viticola lesion associated mycobunyavirales-like virus 8	Discoviridae	Orthodiscovirus	87%	1.00E-89	46.97%	320	QNQ74051.1	283	283
nucleocapsid	Coniothyrium diplodiella negative-stranded RNA virus 1	Discoviridae	Orthodiscovirus	65%	9.00E-85	54.98%	366	QFQ60954.1	272	272
nucleocapsid protein	Coniothyrium diplodiella negative-stranded RNA-like virus 1	Discoviridae	Orthodiscovirus	65%	5.00E-76	47.01%	350	QQO58803.1	249	249
nucleocapsid protein	Penicillium roseopurpureum negative ssRNA virus 1	Discoviridae	Orthodiscovirus	57%	5.00E-67	51.85%	340	QEQ12679.1	226	226
Nucleocapsid	Penicillium discovirus	Discoviridae	Orthodiscovirus	56%	5.00E-47	39.25%	265	AWN00473.1	171	171

A. RBSDV detection

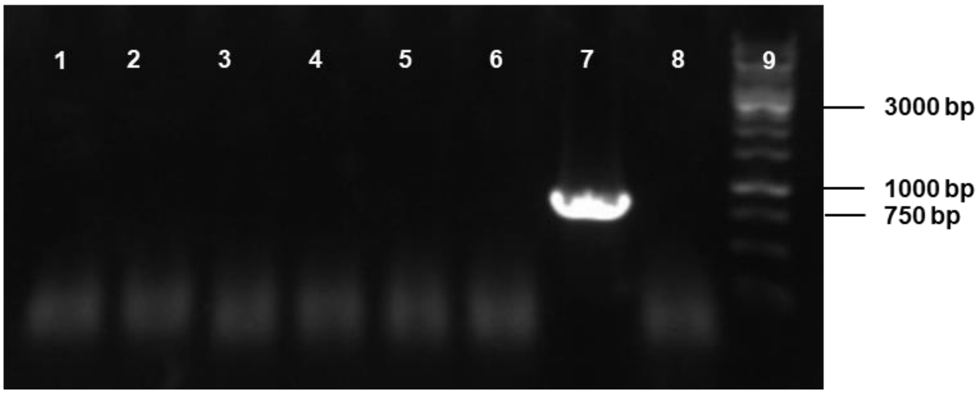

B. SRBSDV detection

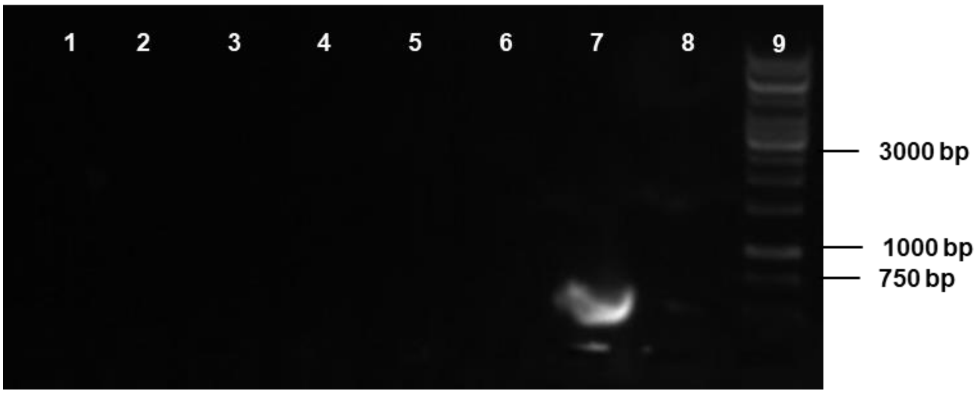

C. RSMV detection

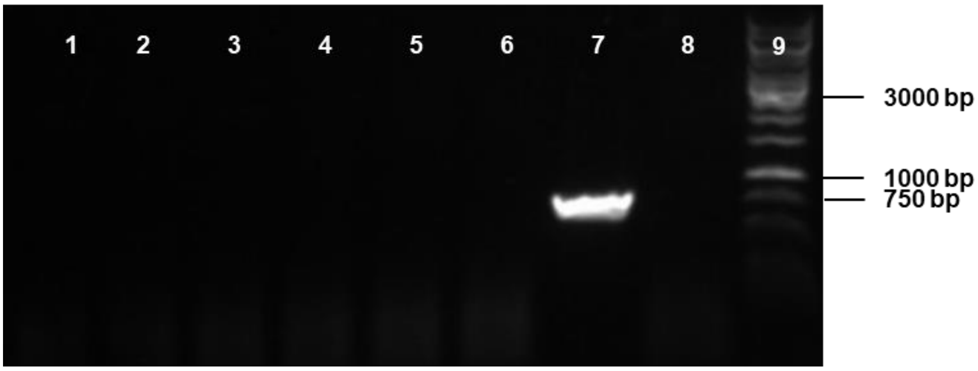

D. RSV detection

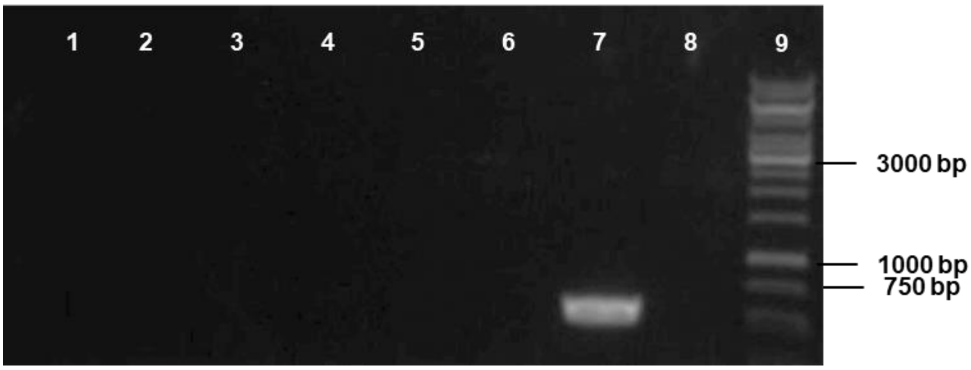

E. RDV detection

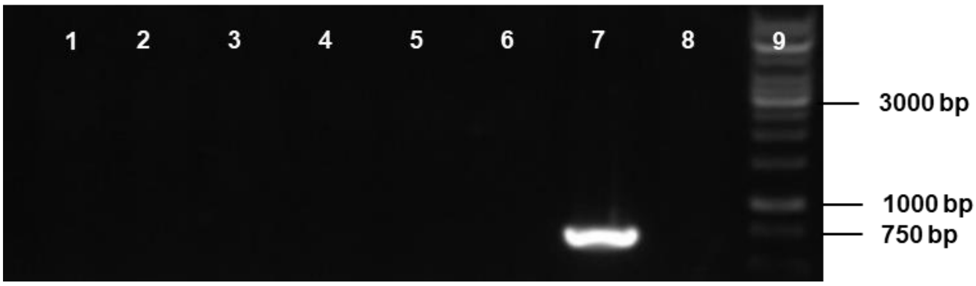

F. RGDV detection

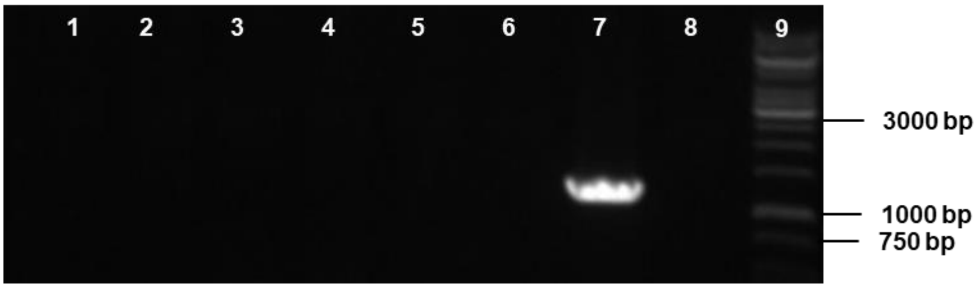

G. RRSV detection

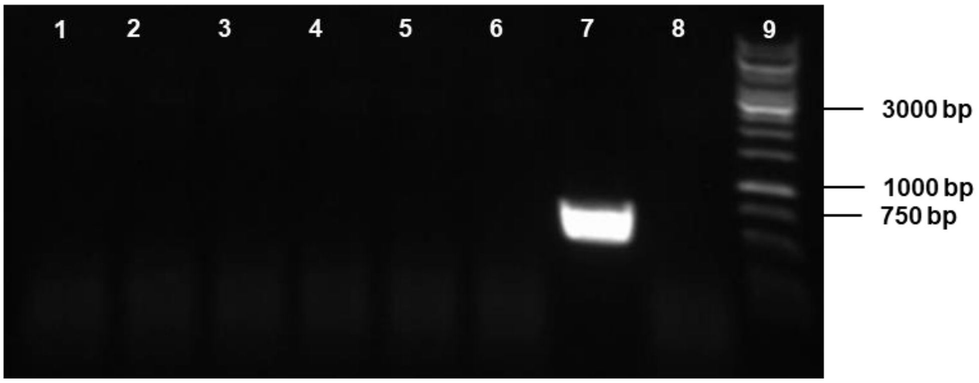

Figure S1. RT-PCR detecting RBSDV (A), SRBSDV (B), RSMV (C) RSV (D), RDV (E), RGDV (F) and RRSV (G) in field-collected rice plants.

Lanes 1-6 were field-collected rice plants as shown in Figure 1A. Lanes 7 and 8 were rice plants infected by the virus and uninfected rice plants, using as positive and negative controls. Lane 9 was a 1 kb DNA marker.

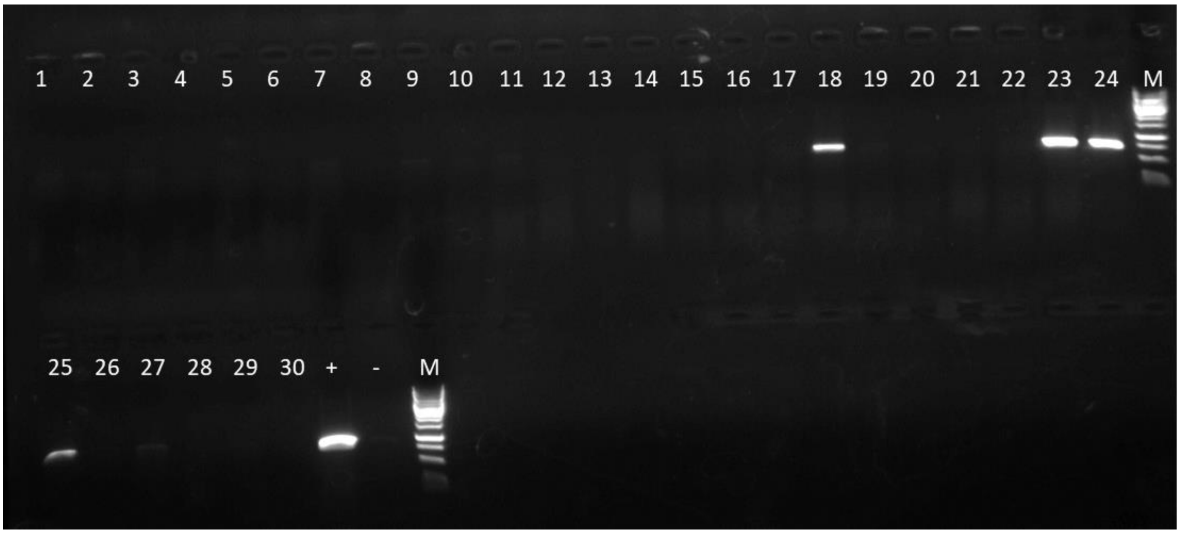

Figure S2. RT-PCR detecting RDaBV in field-collected rice plants.
Lanes 1-30 were 30 rice plant samples collected from paddy fields in Zhejiang Province of China in 2021. + was a RDaBV-infected rice plant. – was an uninfected rice plant. M was a 1 kb DNA marker.
